# Intestinal Absorption and Anti-Inflammatory Effects of a Low-Molecular-Weight α-Glucan from *Flammulina filiformis*

**DOI:** 10.3390/foods15112007

**Published:** 2026-06-04

**Authors:** Yue Li, Mengmeng He, Ziyi Xu, Jianhui Liu, Haoru Zhao, Qiuhui Hu, Fei Pei, Wenjian Yang, Anxiang Su

**Affiliations:** 1 Jiangsu Province Engineering Research Center of Edible Fungus Preservation and Intensive Processing, Nanjing 210023, China; liyue_ayla2001@163.com (Y.L.); 18888537976@163.com (M.H.); xzy2992@163.com (Z.X.); 9120191155@nufe.edu.cn (J.L.); 2120233034@stu.nufe.edu.cn (H.Z.); qiuhuihu@nufe.edu.cn (Q.H.); feipei87@163.com (F.P.); lingwentt@163.com (W.Y.); 2 Collaborative Innovation Center for Modern Grain Circulation and Safety, College of Food Science and Engineering, Nanjing University of Finance and Economics, Nanjing 210023, China

**Keywords:** *Flammulina filiformis* polysaccharide, low-molecular-weight, intestinal absorption, anti-inflammatory effect, metabolomics

## Abstract

The health benefits of *Flammulina filiformis* polysaccharides (FFPs) are often attributed to high-molecular-weight (HMW) fractions, which act via the gut microbiota. In contrast, the direct intestinal absorption and corresponding mechanisms of low-molecular-weight (LMW) FFP fractions are less understood. This study, therefore, focused on isolating an LMW and systematically investigating its absorption and anti-inflammatory potential. Structural characterization identified the LMW-FFP as an α-glucan featuring a backbone of (1→4)- and (1→4,6)-linked α-D-Glcp residues. Transport studies using a Caco-2 cell model showed that fluorescein isothiocyanate (FITC)-labeled LMW-FFP was absorbed across the intestinal epithelium. Mechanistic evidence identified macropinocytosis as the primary pathway. In LPS-induced RAW264.7 macrophages, LMW-FFP (50 μg/mL) significantly inhibited the production of pro-inflammatory mediators (NO, TNF-α, IL-1β, IL-6). Non-targeted metabolomics further suggested that the anti-inflammatory effect was associated with the regulation of histidine and glycerophospholipid metabolism, with histamine as a potential key biomarker. These findings contribute to a more comprehensive understanding of FFP’s structure–activity relationship by highlighting the potential of LMW fractions as directly bioavailable components with anti-inflammatory properties.

## 1. Introduction

*Flammulina filiformis* is a popular edible mushroom renowned for its culinary value and as a rich source of bioactive compounds. Among these, *Flammulina filiformis* polysaccharide (FFP) is recognized as a primary functional constituent, underpinning various health-promoting effects such as antioxidant, immunomodulatory, anti-inflammatory, and hypolipidemic activities [1,2,3,4,5]. These diverse bioactivities highlight the significant potential of FFP in the development of functional foods and nutraceuticals.

To date, research on FFP has predominantly focused on high-molecular-weight (HMW) fractions, which typically exhibit molecular weights ranging from hundreds of thousands to millions of Daltons [6]. HMW polysaccharides generally cannot be absorbed directly across the intestinal epithelium. Consequently, their biological effects arise from indirect mechanisms, primarily via modulation of the gut microbiota and fermentation into short-chain fatty acids and other metabolites [7]. Supporting this notion, studies have demonstrated that FFP intake can enhance the production of acetate, propionate, and butyrate while positively influencing probiotic abundance [8].

Interestingly, however, our previous work revealed that crude FFP (cFFP) extracts still elicited significant immunomodulatory effects in a pseudo-germ-free mouse model [9]. HMW polysaccharides typically act through local intestinal immune recognition or microbiota-dependent fermentation. In contrast, this finding suggests that a low-molecular-weight (LMW) fraction with favorable intestinal permeability exists. Such LMW components may have the potential to be directly absorbed into the systemic circulation, thereby interacting with target cells beyond the gastrointestinal tract.

Despite this compelling evidence, critical knowledge gaps regarding LMW-FFP remain. First, the structure of purified LMW-FFP remains incompletely characterized, including its glycosidic linkages, monosaccharide sequence, and conformation [10]. Second, the intestinal absorption mechanism of LMW-FFP is unclear. While the microbiota-dependent pathway of HMW-FFP is partially established, direct evidence for the uptake of LMW-FFP by intestinal epithelial cells and the specific transport mechanisms involved is underexplored [11]. Although some studies have reported that fungal polysaccharides from sources like Ganoderma lucidum can be absorbed by Caco-2 cells via macropinocytosis, the specific uptake pathways and systemic metabolic impacts of LMW-FFP from *Flammulina filiformis* remain largely unknown [12]. Finally, the direct anti-inflammatory mechanism of absorbable LMW-FFP is poorly understood. Although the anti-inflammatory activity of FFP is well-documented [13,14], most studies emphasize microbiota-mediated indirect effects. Research investigating the direct immunomodulatory effects of LMW-FFP on immune cells like macrophages and the underlying metabolic mechanisms remains scarce [15].

To address these research gaps, the present study was designed with the following objectives: (1) to isolate and purify a LMW-FFP fraction using ultrasound-assisted extraction followed by column chromatography, and to systematically characterize its structure using integrated techniques including chromatography, spectroscopy, and nuclear magnetic resonance (NMR); (2) to investigate the intestinal absorption behavior and transport pathways of LMW-FFP employing a fluorescein isothiocyanate (FITC)-labeling strategy and a Caco-2 cell monolayer model; and (3) to evaluate the direct anti-inflammatory effects of LMW-FFP in lipopolysaccharide (LPS)-induced RAW264.7 macrophages and to uncover the potential metabolic mechanisms using LC-MS-based non-targeted metabolomics. Based on the prior understanding that neutral polysaccharides are more amenable to direct intestinal absorption compared to their acidic counterparts, this study initially focused on the neutral water-eluted fraction obtained through ion-exchange chromatography.

This research aims to provide a theoretical foundation for elucidating the direct biological functions of LMW-FFP, thereby facilitating the targeted development of high-value, bioavailable functional ingredients derived from *Flammulina filiformis*.

## 2. Materials and Methods

### 2.1. Materials and Reagents

DEAE Sepharose Fast Flow was purchased from Cytiva (Uppsala, Sweden). Monosaccharide standards (purity ≥ 98%), 1-Phenyl-3-methyl-5-pyrazolone (PMP, ≥98%), and Fluorescein isothiocyanate (FITC, ≥90%) were obtained from Yuanye Bio-technology Co., Ltd. (Shanghai, China). Dextran standards (1–670 kDa) and Fluorescein sodium (≥95%) were procured from Sigma-Aldrich (St. Louis, MO, USA). Dulbecco’s Modified Eagle Medium (DMEM), fetal bovine serum (FBS), penicillin–streptomycin, and trypsin-EDTA were purchased from Gibco (Thermo Fisher Scientific, Waltham, MA, USA). All other chemicals and solvents were of analytical grade.

### 2.2. Extraction, Purification, and Physicochemical Characterization of FFP

#### 2.2.1. Preparation of Crude Polysaccharides

Freeze-dried *Flammulina filiformis* powder (60 g) was defatted with 85% ethanol (1:15, *w*/*v*) at room temperature for 12 h. The pellet was collected by centrifugation at 3000× *g* for 12 min using a TGL-16G refrigerated centrifuge (Hunan Hexi Instrument Equipment Co., Ltd., Changsha, China) and subjected to ultrasound-assisted extraction with deionized water (1:30, *w*/*v*) at 45 °C for 50 min (620 W). These extraction parameters were optimized based on preliminary single-factor experiments to achieve maximum yield while minimizing potential polysaccharide degradation caused by excessive thermal or ultrasonic energy. Specifically, the moderate temperature of 45 °C and the ultrasonic power of 620 W were selected to balance extraction efficiency with structural integrity, as higher energy levels were observed to significantly reduce molecular weight. The supernatant was concentrated under reduced pressure at 55 °C using a rotary evaporator (Hei-VAP Expert, Heidolph Instruments GmbH, Schwabach, Germany), and polysaccharides were precipitated with three volumes of 95% ethanol at 4 °C overnight. The precipitate was collected, redissolved in water, and deproteinized using Sevag reagent (chloroform: n-butanol = 4:1, *v*/*v*). The resulting aqueous solution was dialyzed (MWCO 3.5 kDa) and lyophilized to obtain crude FFP (cFFP).

#### 2.2.2. Purification by Ion-Exchange and Gel-Filtration Chromatography

The cFFP was fractionated on a DEAE Sepharose Fast Flow column (3.0 × 50 cm, Cytiva, Uppsala, Sweden) eluted with ultrapure water. The water-eluted neutral fraction was collected, concentrated, and further purified on a Chromdex 75PG column (2.6 × 100 cm, Bestchrom, Jiaxing, China) eluted with ultrapure water at 1.5 mL/min. The major symmetrical peak was collected, dialyzed (MWCO 3.5 kDa), and lyophilized to obtain the purified polysaccharide FFP.

#### 2.2.3. Molecular Weight and Monosaccharide Composition Analysis

The average molecular weight of FFP was determined by HPGPC using dextran standards. Monosaccharide composition was analyzed by HPLC after acid hydrolysis (2 M TFA, 121 °C, 2 h) and derivatization with PMP. This hydrolysis condition is widely accepted for ensuring complete cleavage of glycosidic linkages in inulin-type fructans, as demonstrated in previous structural studies [16]. The analysis was performed on an Agilent Eclipse XDB-C18 column with a mobile phase of acetonitrile and phosphate buffer (pH 6.8) (17:83, *v*/*v*) at 0.8 mL/min.

### 2.3. Structural Characterization of FFP

#### 2.3.1. Fourier Transform Infrared (FT-IR) Spectroscopy

The FT-IR spectrum of FFP was recorded using a KBr pellet method on an IRAffinity-1S spectrometer (Shimadzu, Kyoto, Japan) over the range of 4000–400 cm^−1^.

#### 2.3.2. Methylation Analysis

FFP was methylated three times using the Ciucanu and Kerek method with methyl iodide and NaOH in DMSO. The methylated product was hydrolyzed with 2 M TFA at 121 °C for 2 h, reduced with NaBD_4_, and acetylated with acetic anhydride to produce partially methylated alditol acetates (PMAAs). The PMAAs were analyzed by GC-MS (7890B-5977B; Agilent Technologies, Santa Clara, CA, USA) using an HP-5MS capillary column (30 m × 0.25 mm × 0.25 μm). The temperature program was as follows: initial 120 °C for 2 min, ramped to 250 °C at 5 °C/min, and held for 5 min. Helium was used as the carrier gas at a flow rate of 1 mL/min. Identification of PMAAs was based on retention times and mass spectral comparison with the CCRC Spectral Database for PMAAs.

#### 2.3.3. Nuclear Magnetic Resonance (NMR) Spectroscopy

FFP (30 mg) was dissolved in D_2_O and analyzed using an AVANCE III HD NMR spectrometer (Bruker Biospin, Fällanden, Switzerland). ^1^H NMR, ^13^C NMR, and two-dimensional spectra (COSY, HSQC, HMBC) were acquired at 25 °C.

### 2.4. Intestinal Absorption Studies Using a Caco-2 Cell Model

#### 2.4.1. Fluorescent Labeling of FFP (FFFP)

FFP was labeled with FITC via a tyramine linker. Briefly, FFP was conjugated with tyramine in an alkaline buffer, followed by reaction with FITC. The FITC-labeled FFP (FFFP) was purified by dialysis and characterized by UV-Vis spectroscopy (UV-2600; Shimadzu, Kyoto, Japan).

#### 2.4.2. Cell Culture and Cytotoxicity Assay

Caco-2 cells were cultured in DMEM supplemented with 10% FBS. The cytotoxicity of FFFP was evaluated using the CCK-8 assay after 24 h of exposure.

#### 2.4.3. Transport Studies

Caco-2 cells were seeded on Transwell inserts and cultured for 21 days until TEER values exceeded 300 Ω·cm^2^. The transport of FFFP (10–50 μg/mL) across the monolayer was investigated in both apical-to-basolateral (AP→BL) and basolateral-to-apical (BL→AP) directions. The apparent permeability coefficient (Papp) was calculated. To explore the transport mechanism, cells were pre-incubated with specific inhibitors (amiloride, chlorpromazine, genistein, verapamil). The concentrations of these inhibitors (chlorpromazine 20 µM, genistein 60 µM, amiloride 100 µM, and verapamil 100 µM) were pre-determined by CCK-8 assays, which confirmed that these concentrations had no significant cytotoxicity on Caco-2 cells (cell viability > 95%) during the experimental period, ensuring that the observed inhibitory effects were specifically due to the blockade of transport pathways.

### 2.5. In Vitro Anti-Inflammatory Activity and Metabolomics

#### 2.5.1. Cell Culture and Viability Assay

RAW264.7 macrophages were cultured in DMEM. The cytotoxicity of FFP was assessed using the CCK-8 assay.

#### 2.5.2. Anti-Inflammatory Activity Assessment

Cells were pre-treated with FFP (10, 25, 50 μg/mL) for 4 h, followed by stimulation with LPS (0.5 μg/mL) for 24 h. The levels of NO (Griess reagent) and pro-inflammatory cytokines (TNF-α, IL-1β, IL-6; ELISA) in the culture supernatant were measured.

#### 2.5.3. Cellular Metabolomics Analysis

After treatment (Control, LPS, LPS + FFP), cells were quenched, and metabolites were extracted with cold methanol–water (4:1, *v*/*v*) containing an internal standard. Metabolite profiling was performed using a UHPLC system coupled with a Q-Exactive mass spectrometer (Thermo Fisher Scientific, Waltham, MA, USA) in both positive and negative ionization modes. Data were processed using appropriate software for multivariate statistical analysis.

### 2.6. Statistical Analysis

All experiments were performed in at least triplicate (n = 3, independent experiments). Data are presented as mean ± standard deviation (SD). Statistical significance was determined by one-way analysis of variance (ANOVA) followed by Duncan’s test using SPSS 26.0 (IBM, Chicago, IL, USA). Duncan’s multiple range test was selected to provide higher statistical power for detecting subtle yet biologically relevant differences among the multiple concentration groups. The effect size (η^2^) and 95% confidence intervals (CI) were calculated to evaluate the magnitude and precision of the observed effects, respectively. A *p* < 0.05 was considered significant.

## 3. Results and Discussion

### 3.1. Isolation, Purification, and Structural Elucidation of a Low-Molecular-Weight FFP

**Figure 1:**
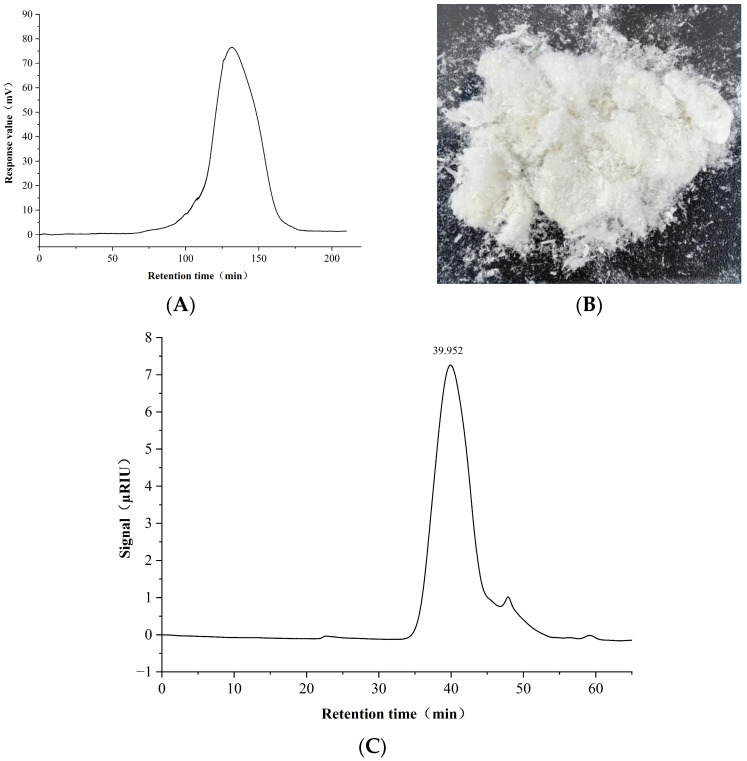
Purification and molecular weight characterization of FFP (**A**); Sample image of low-molecular-weight polysaccharide from *Flammulina filiformis* (**B**); Photograph of lyophilized FFP powder (**C**).

To investigate whether FFP has direct biological effects independent of the gut microbiota, we undertook the isolation of its low-molecular-weight (LMW) neutral fraction, which we hypothesized would have enhanced absorbability. The crude polysaccharides were initially fractionated by anion-exchange chromatography on a DEAE Sepharose Fast Flow column. As anticipated for a neutral polysaccharide, a single, symmetrical peak was obtained upon elution with ultrapure water (Figure 1A), indicating the absence of strong electrostatic interaction with the anion-exchange medium. This water-eluted fraction was collected as the target neutral polysaccharide for subsequent purification.

Further purification of this neutral fraction was achieved using gel-filtration chromatography on a Chromdex 75PG column. The high-performance liquid chromatography (HPLC) profile displayed a single, symmetric peak (Figure 1B), suggesting the homogeneity of the purified polysaccharide. The corresponding fraction was collected, yielding a white, fluffy powder after lyophilization (Figure 1C). High-performance gel permeation chromatography (HPGPC) analysis confirmed the homogeneity of the final product, showing a single and symmetric peak. Based on the calibration curve established with dextran standards, the average molecular weight (Mw) of this purified polysaccharide was calculated to be 6.2 kDa, and it was designated FFP for further study.

The molecular weight of 6.2 kDa is a critical finding, as it is substantially lower than the high-molecular-weight (HMW) fractions (typically ranging from 10^4^ to 10^5^ Da) commonly reported for *Flammulina filiformis* polysaccharides extracted by conventional hot-water methods [17]. This reduction in molecular weight likely resulted from the ultrasonic-assisted extraction process used in this study. It is well-documented that the powerful cavitation effects generated by ultrasound irradiation can effectively cleave glycosidic bonds within polysaccharide chains, leading to molecular weight degradation [17,18]. The successful isolation of this LMW fraction is particularly relevant because smaller polysaccharides have been demonstrated to exhibit superior intestinal absorption efficiency compared to their HMW counterparts [19]. Thus, the acquisition of a 6.2 kDa FFP provides a fundamental and suitable material basis for our subsequent investigation into its direct intestinal absorption mechanism and associated bioactivities.

**Figure 2:**
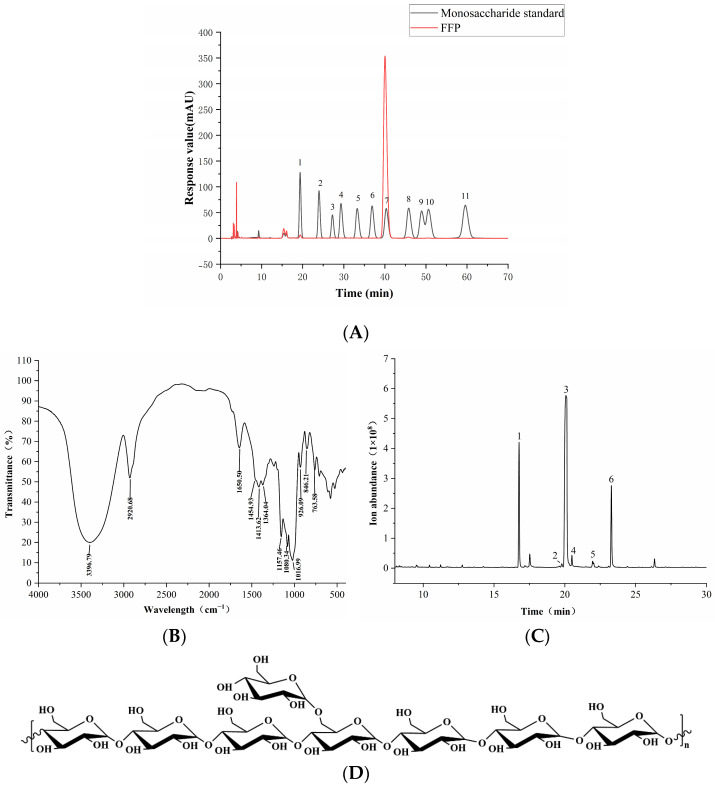
HPLC chromatogram of monosaccharide composition of mixed standard and FFP purified component (**A**), 1. Mannose; 2. Glucosamine hydrochloride; 3. Rhamnose; 4. Glucuronic acid; 5. Galacturonic acid; 6. N-acetylgalactosamine hydrochloride; 7. Glucose; 8. Galactose; 9. Xylose; 10. Arabinose; 11. Fucose; FT-IR spectrum of FFP (**B**); The total ion chromatogram of FFP (**C**); The molecular structure of FFP (**D**).

Structural characterization commenced with monosaccharide composition analysis. The results indicated that FFP was predominantly composed of glucose (98.87 mol%), with only trace amounts of mannose and galactose (Figure 2A). This unambiguously identifies FFP as a glucan. While glucose is a major component in many reported *Flammulina filiformis* polysaccharides, the exceptionally high purity and relatively low molecular weight of our FFP distinguish it from the more commonly reported HMW, heteropolymeric fractions [14]. This structural specificity likely stems from our tailored extraction and purification strategy, which selectively enriched a low-molecular-weight, neutral α-glucan fraction.

Fourier-transform infrared (FT-IR) spectroscopy provided further insights into the functional groups and glycosidic linkage types of FFP (Figure 2B). The characteristic broad absorption peak at around 3396 cm^−1^ was assigned to O-H stretching vibrations. The weak C-H stretching band was observed at 2920 cm^−1^. Crucially, the absorption peak at approximately 846 cm^−1^ is a characteristic signal of α-type glycosidic linkages [20], while the absence of a peak near 890 cm^−1^ (typical for β-configurations) supports this assignment. The intense absorptions between 1200 and 1000 cm^−1^ are characteristic of pyranose rings.

To determine the detailed linkage patterns, FFP was subjected to methylation analysis followed by GC-MS (7890B-5977B; Agilent Technologies, Santa Clara, CA, USA). The results (Figure 2C and Table 1) revealed that FFP primarily consisted of 1,4-linked-Glcp (67.20%), along with terminal-Glcp (18.75%), 1,4,6-linked-Glcp (11.31%), and minor amounts of 1,6-linked-Glcp and 1,3,4-linked-Glcp. This linkage pattern confirms a backbone primarily built of (1→4)-linked glucose residues, with branching points at the O-6 position.

**Figure 3:**
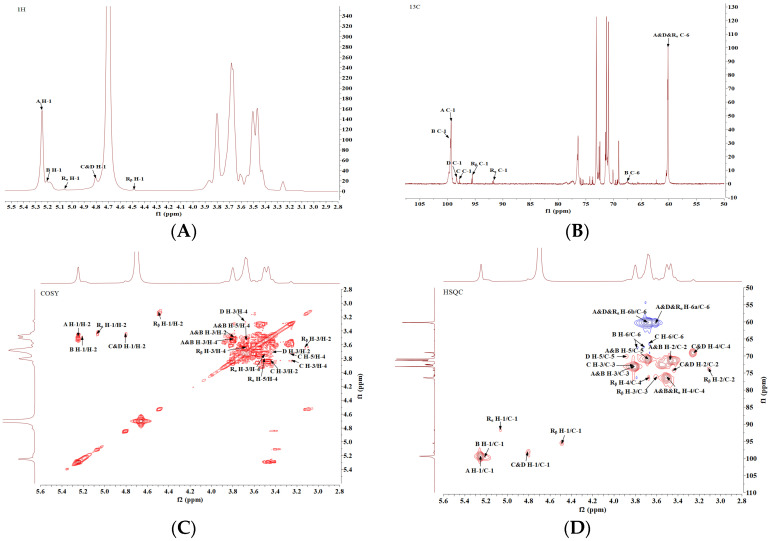
One-dimensional and basic two-dimensional NMR spectra of FFP dissolved in D_2_O at 25 °C. ^1^H NMR (**A**), ^13^C NMR (**B**), COSY (**C**), HSQC (**D**).

**Figure 4:**
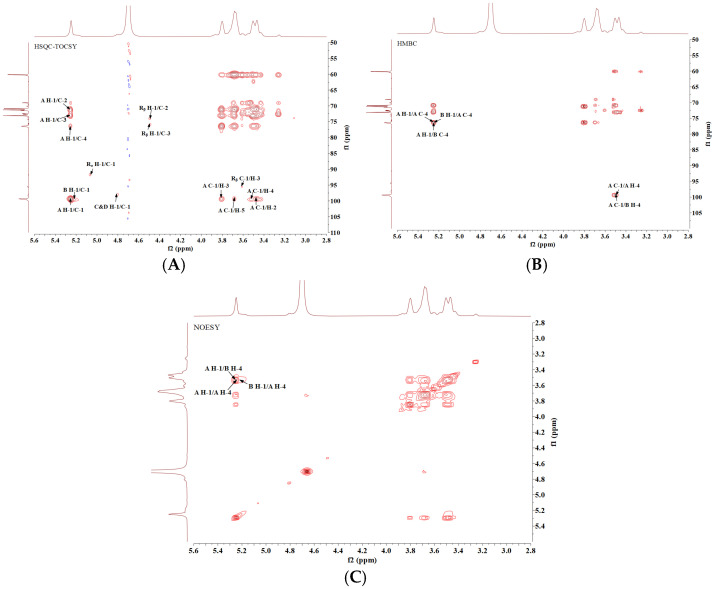
Advanced two-dimensional NMR spectra for linkage sequence elucidation of FFP: HSQC-TOCSY (**A**), HMBC (**B**), and NOESY (**C**).

The structure was further elucidated by one-dimensional (^1^H, ^13^C) and two-dimensional (COSY, HSQC, HMBC, NOESY) NMR spectroscopy (Figure 2D, Figure 3A–D and Figure 4A–C, and Table 2). The NMR data were fully consistent with the methylation results. The anomeric proton signals in the region of δ 4.8–5.3 ppm and corresponding carbon signals around δ 97–100 ppm confirmed the α-configuration. Detailed analysis allowed the identification of four main residue types: →4)-α-D-Glcp-(1→ (Residue A), →4,6)-α-D-Glcp-(1→ (Residue B), →6)-α-D-Glcp-(1→ (Residue C), and α-D-Glcp-(1→ (Residue D). Critically, the key cross-signals observed in the HMBC and NOESY spectra between the anomeric proton of Residue A and H4/C4 of Residue B (and vice versa) unambiguously demonstrated that the backbone consists of alternating (1→4)- and (1→4,6)-linked α-D-glucopyranose residues (Figure 4B,C). The proposed structure of FFP is summarized in Figure 2D.

The elucidated structure, particularly the backbone rich in (1→4)-linked α-D-Glcp residues, is of significant biological relevance. Such (1→4)-α-glucan structures have been frequently implicated in the anti-inflammatory and immunomodulatory activities of polysaccharides from various botanical and fungal sources [21,22]. For instance, similar structural motifs are found in known bioactive glucans. Therefore, the precise structural features of FFP not only define its chemical identity but also provide a strong molecular basis for hypothesizing its potential direct anti-inflammatory effects, which we sought to validate in subsequent cellular assays independent of gut microbiota.

### 3.2. Intestinal Absorption of LMW-FFP Occurs via Macropinocytosis

**Figure 5:**
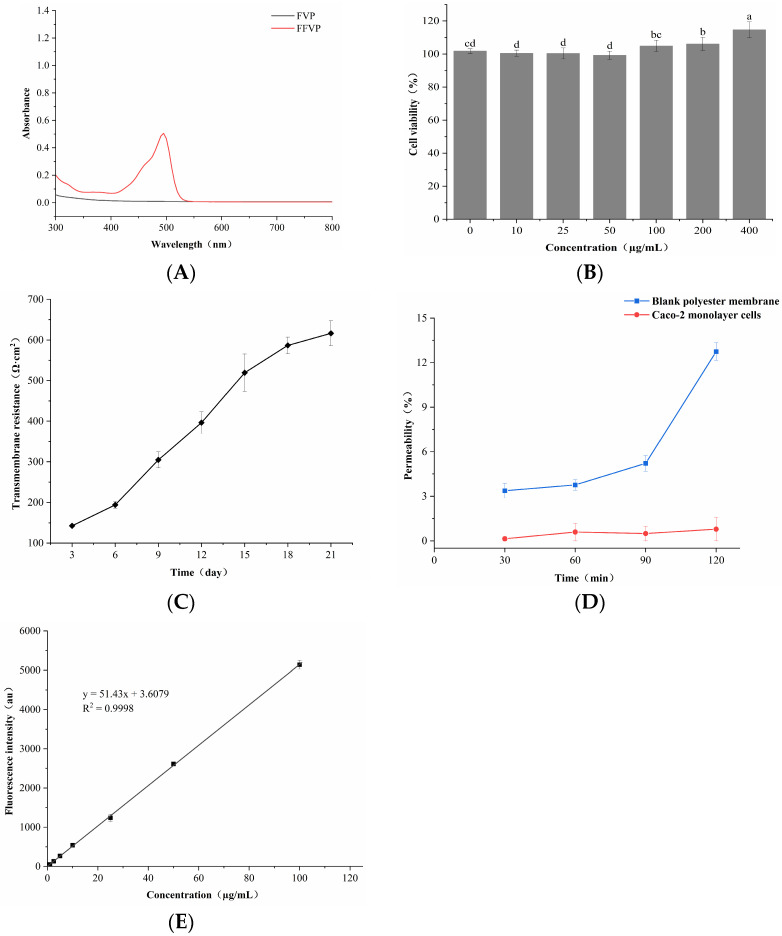
The Ultraviolet–Visible spectrum of FFP and FFFP (**A**); The effect of different concentrations of FFFP on cell viability of Caco-2 cells (**B**); Monolayer transmembrane resistance of Caco-2 cells (**C**); The permeability of fluorescein sodium in blank polyester membrane and Caco-2 cell monolayer at different time points (**D**); The standard curve of FFFP fluorescence intensity (**E**). Data are expressed as the mean ± standard deviation (SD). For subfigure (**B**), different lowercase letters (a–d) above the bars indicate significant differences between groups (*p* < 0.05) based on Duncan’s multiple range test.

Having established the precise structure of the low-molecular-weight FFP, we next sought to investigate its potential for direct intestinal absorption, a prerequisite for its hypothesized direct systemic effects. To enable real-time tracking, LMW-FFP was successfully labeled with fluorescein isothiocyanate (FITC) via a tyramine linker, yielding the fluorescent derivative FFFP. UV-Vis spectroscopy confirmed the successful conjugation, as evidenced by the characteristic absorption peak of FITC at 490 nm for FFFP, which was absent in the native LMW-FFP spectrum (Figure 5A). The degree of fluorescence substitution was 0.6%, which is unlikely to alter the physicochemical properties of the polysaccharide [23]. Prior to transport studies, the biocompatibility of FFFP with intestinal epithelial cells was assessed. The CCK-8 assay demonstrated that FFFP exhibited no cytotoxicity to Caco-2 cells at concentrations up to 400 μg/mL over 24 h; indeed, cell viability remained at or above 100% (Figure 5B). This confirms that the fluorescent labeling process did not introduce cytotoxic effects and that subsequent experiments could be conducted within a safe concentration range.

The intestinal absorption potential was evaluated using a well-established in vitro model of the intestinal barrier: the Caco-2 cell monolayer [24]. Caco-2 cells were cultured on Transwell inserts for 21 days to allow for full differentiation and the formation of tight junctions. The integrity of the monolayers was rigorously monitored by measuring the transepithelial electrical resistance (TEER). TEER values increased steadily over time, stabilizing at approximately 616 Ω·cm^2^ by day 21 (Figure 5C), indicating the formation of a robust and confluent epithelial barrier. This barrier functionality was further validated by a sodium fluorescein permeability assay. The permeability of sodium fluorescein (Mw: 376 Da) through the cell monolayer was significantly lower than through the blank insert membrane, increasing only marginally from 0.2% to 1.6% over 2 h (Figure 5D). This low permeability, consistent with the high TEER values, confirmed that the monolayers were functionally intact and suitable for studying macromolecular transport [25,26].

**Figure 6:**
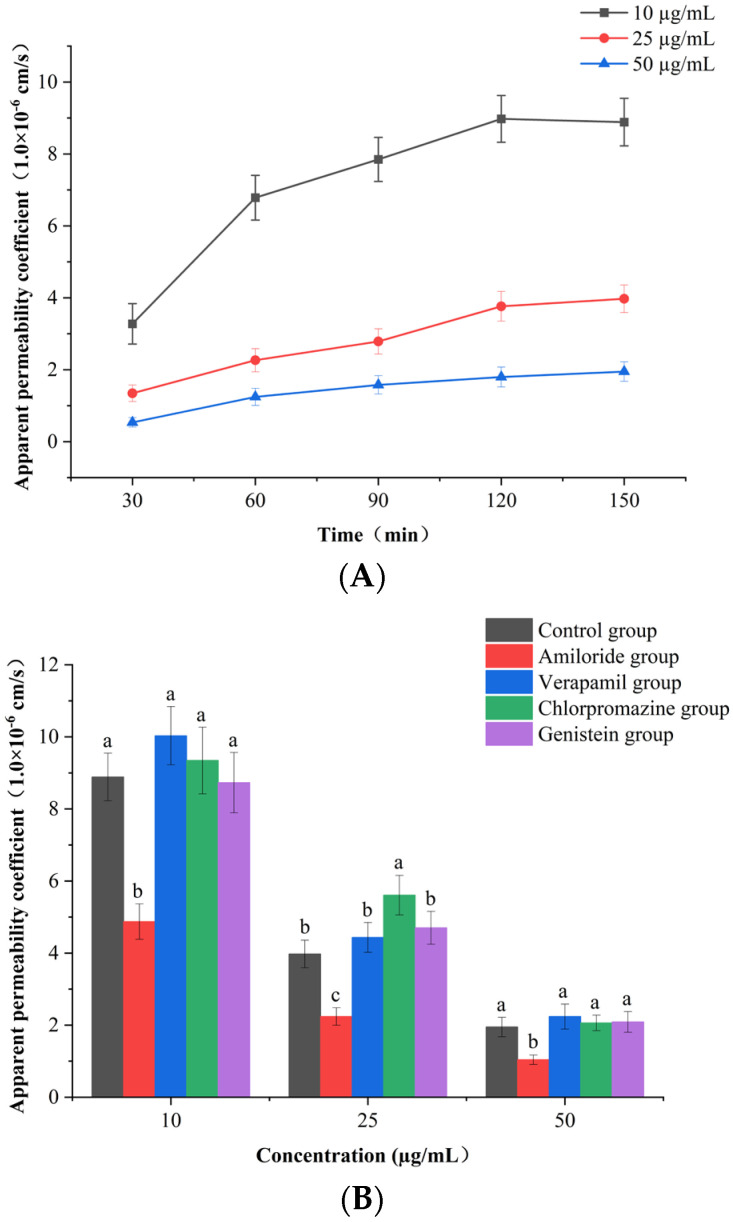
Papp value of FFFP with different concentrations from AP to BL at different times (**A**); Effect of four transport inhibitors on the apparent permeability of FFFP (**B**). Data are expressed as the mean ± standard deviation (SD). For subfigure (**B**), different lowercase letters (a–c) above the bars indicate significant differences between groups (*p* < 0.05) based on Duncan’s multiple range test.

The transmembrane transport of FFFP was first examined in the apical-to-basolateral (AP→BL) direction, simulating absorption from the intestinal lumen into the bloodstream. A standard curve of FFFP fluorescence intensity versus concentration was established with excellent linearity (y = 51.43x + 3.6079, R^2^ = 0.9998, Figure 5E). The transport experiments revealed that FFFP was able to traverse the Caco-2 monolayer in a time-dependent manner. Notably, the apparent permeability coefficient (Papp) decreased with increasing initial FFFP concentration (from 10 to 50 μg/mL), yielding values of 8.89 × 10^−6^, 3.97 × 10^−6^, and 1.95 × 10^−6^ cm/s, respectively (Figure 6A, Table 3). This inverse relationship between Papp and concentration can be indicative of a saturable transport process, such as receptor-mediated or energy-dependent endocytosis, as opposed to simple passive diffusion. The Papp values obtained place FFFP in the category of moderately absorbed compounds, providing strong initial evidence that this LMW polysaccharide can indeed cross the intestinal epithelium.

To elucidate the specific cellular pathway responsible for FFP uptake, we employed a pharmacological inhibitor approach. Caco-2 monolayers were pre-incubated with specific inhibitors targeting various major endocytic and efflux pathways: chlorpromazine (clathrin-mediated endocytosis inhibitor), genistein (caveolae-mediated endocytosis inhibitor), amiloride (macropinocytosis inhibitor), and verapamil (P-glycoprotein efflux transporter inhibitor). The results were striking. Pre-treatment with amiloride (100 μM) significantly reduced the Papp value of FFFP by approximately 55% compared to the control group (Figure 6B). In contrast, chlorpromazine, genistein, and verapamil showed no significant inhibitory effects. Furthermore, the efflux ratio (ER), calculated from the Papp values in the BL→AP and AP→BL directions, was consistently less than 1 across all tested concentrations (Table 3), effectively ruling out the involvement of active efflux transporters like P-gp.

Collectively, this pattern of inhibition—specific suppression by amiloride and no effect from inhibitors of other pathways—provides compelling evidence that the intestinal absorption of LMW FFP occurs primarily via macropinocytosis. Macropinocytosis is a non-selective, actin-dependent form of endocytosis that mediates the bulk uptake of extracellular fluid and solutes, making it a plausible mechanism for the internalization of a 6.2 kDa polysaccharide [25]. This finding is significant because it distinguishes the absorption mechanism of FFP from the paracellular transport of very small molecules or the highly specific receptor-mediated endocytosis of certain ligands. It confirms that certain LMW fungal polysaccharides can be directly taken up by intestinal epithelial cells through a defined cellular process, bypassing the prerequisite for microbial fermentation. This discovery provides a crucial mechanistic foundation for understanding how directly absorbable polysaccharides can exert systemic biological effects.

The concentration-dependent decrease in Papp values suggests that the transport of LMW-FFP is a saturable process. This is mechanistically supported by the significant inhibitory effect of amiloride, confirming macropinocytosis as the primary internalization pathway. Furthermore, the lack of influence from verapamil excludes P-gp-mediated efflux, ensuring efficient transcellular transport. Collectively, these results align with recent observations that structurally similar fungal polysaccharides utilize macropinocytosis for intestinal crossing, establishing a plausible framework for their direct systemic bioavailability [27].

### 3.3. FFP Directly Attenuates LPS-Induced Inflammation in Macrophages

Having established that LMW FFP can cross the Caco-2 intestinal epithelial monolayer via macropinocytosis, it is crucial to investigate its subsequent biological targets. Once absorbed into the lamina propria or systemic circulation, macrophages are among the primary immune cells that these polysaccharides would encounter. Therefore, to ensure biological relevance, we utilized the RAW264.7 macrophage model to evaluate the potential direct anti-inflammatory mechanisms of the absorbed FFP. Macrophages regulate the initiation and resolution of inflammation, and their dysfunction contributes to many chronic diseases. We, therefore, evaluated the direct anti-inflammatory activity of FFP using the murine macrophage cell line RAW264.7.

**Figure 7:**
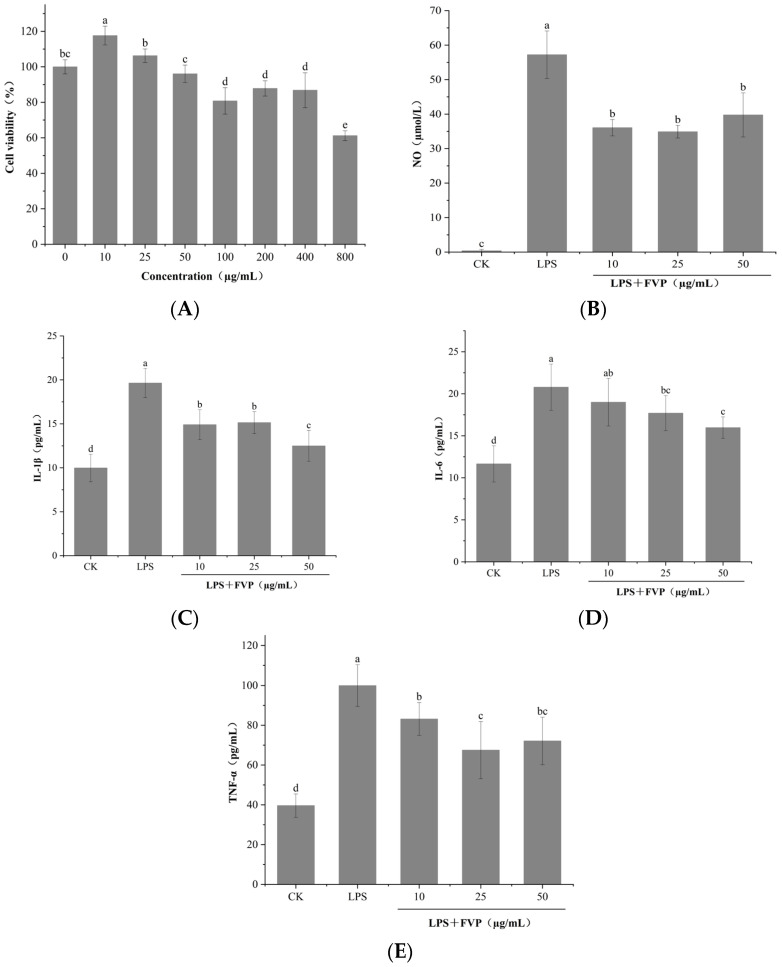
The effect of different concentrations of FFP on cell viability of RAW264.7 cells (**A**); The effect of different concentrations of FFP on NO (**B**), IL-1β (**C**), IL-6 (**D**), and TNF-α (**E**) production of LPS-induced RAW264.7 cells; Data are expressed as mean ± SD (n = 3). Different lowercase letters (a–e) above the bars indicate significant differences between groups (*p* < 0.05) based on one-way ANOVA followed by Duncan’s test. The effect size (η^2^ ≈ 0.85) and 95% CI (calculated for the 50 μg/mL group) confirm the magnitude and precision of the observed effects.

To ensure that any observed effects were not due to cytotoxicity, we first assessed the impact of FFP on RAW264.7 cell viability using the CCK-8 assay. Treatment with FFP at concentrations ranging from 10 to 50 μg/mL for 24 h did not significantly affect cell viability, with survival rates remaining above 100% compared to the control group (Figure 7A). This defined a non-toxic concentration range for subsequent anti-inflammatory experiments. In contrast, concentrations of 100 μg/mL and higher induced a significant decrease in cell viability, underscoring the importance of dose selection in functional studies.

To evaluate the anti-inflammatory efficacy of FFP, we employed a well-established model of inflammation by stimulating RAW264.7 cells with bacterial lipopolysaccharide (LPS). LPS activates Toll-like receptor 4 (TLR4). This triggers a downstream signaling cascade that activates nuclear factor-kappa B (NF-κB), leading to the production of pro-inflammatory mediators [28]. Cells were pre-treated with non-toxic concentrations of FFP (10, 25, and 50 μg/mL) for 4 h before LPS (0.5 μg/mL) challenge. As expected, LPS stimulation significantly induced the production of key inflammatory mediators, including nitric oxide (NO), tumor necrosis factor-alpha (TNF-α), interleukin-1 beta (IL-1β), and interleukin-6 (IL-6), compared to the unstimulated control group (Figure 7B–E).

Crucially, pre-treatment with FFP significantly suppressed this LPS-induced inflammatory response in a clear dose-dependent manner. At the lowest concentration (10 μg/mL), FFP already demonstrated a significant inhibitory effect on the release of NO, TNF-α, and IL-1β. At 25 μg/mL, FFP significantly suppressed the production of all four mediators investigated. The most potent effect was observed at 50 μg/mL FFP, which strongly inhibited the levels of NO, TNF-α, IL-1β, and IL-6, with the suppression of IL-1β and IL-6 being particularly pronounced compared to the lower concentrations (Figure 7C,D). The potency of FFP at 50 μg/mL is comparable to, or even superior to, effective concentrations reported for other bioactive polysaccharides from fungi and plants [23].

Most importantly, these anti-inflammatory effects were observed in a macrophage cell line cultured in the absence of gut microbiota or their metabolites. This provides compelling in vitro evidence that FFP exerts a direct anti-inflammatory action on immune cells. This finding is significant because it demonstrates that, unlike HMW dietary polysaccharides that often rely on local intestinal immune recognition or microbiota-dependent fermentation [29], absorbable LMW polysaccharides like FFP possess the potential to act systemically. Once entering the systemic circulation, they can directly modulate target immune cells, thereby expanding the spatial scope of their biological action.

The dose-dependent inhibition of NO and pro-inflammatory cytokines (TNF-α, IL-1β, IL-6) strongly suggests that FFP may interfere with a key upstream signaling pathway common to their expression, such as the NF-κB pathway. This hypothesis is consistent with the action of many anti-inflammatory compounds and provides a focused direction for future mechanistic studies into the signaling pathways modulated by FFP [10,11,13].

### 3.4. Metabolomic Profiling Reveals the Anti-Inflammatory Mechanism of FFP via Regulation of Histamine and Glycerophospholipid Metabolism

To gain deeper insights into the molecular mechanisms underlying the direct anti-inflammatory effects of FFP, we employed a non-targeted metabolomics approach. This hypothesis-free strategy allows for the global profiling of cellular metabolites, enabling the identification of key metabolic pathways perturbed by LPS-induced inflammation and, more importantly, modulated by FFP intervention.

**Figure 8:**
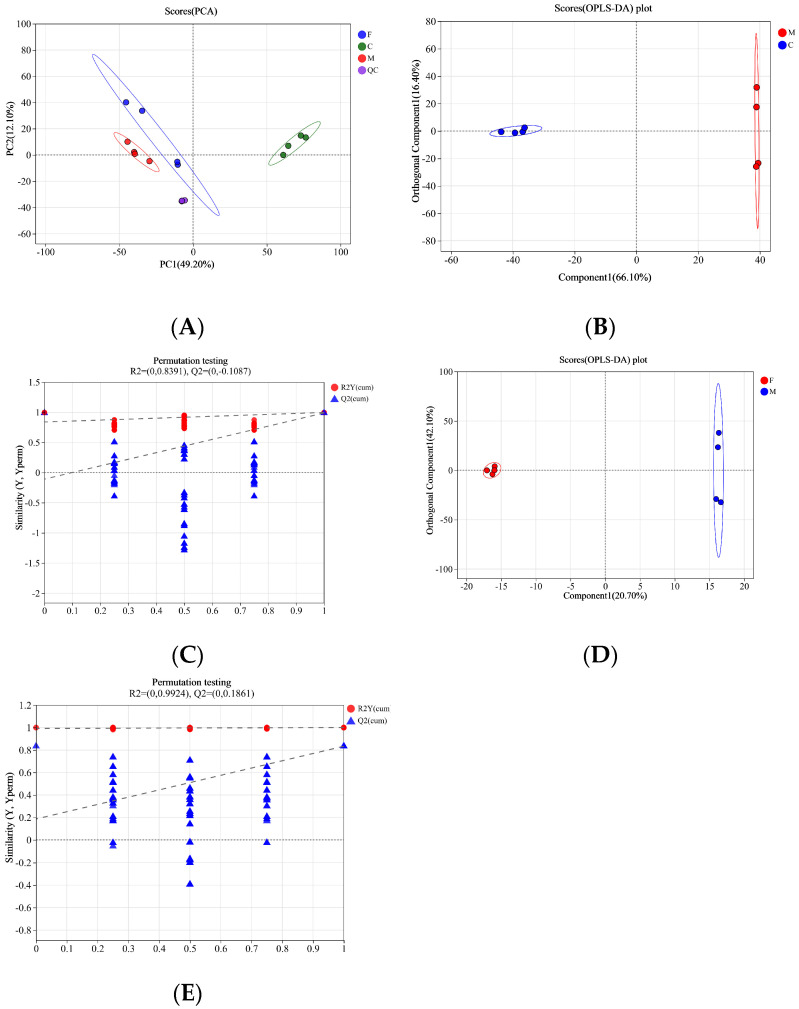
Metabolomics analysis of RAW264.7 macrophages. (**A**) PCA score plot of metabolic profiles across control, LPS-model, and FFP-treated groups. (**B**) OPLS-DA score plot between control and model groups. (**C**) Permutation test (n = 200) for the OPLS-DA model in (**B**); the negative Q^2^ intercept indicates no overfitting. (**D**) OPLS-DA score plot between model and FFP-treated groups. (**E**) Permutation test (n = 200) for the OPLS-DA model in (**D**). Note: Due to the original data format provided by the analytical service, the minus sign symbols and labels represent the raw data output and could not be further modified. We have ensured that these labels are consistent with the data interpretation provided in the main text.

RAW264.7 macrophages were treated under three conditions: control (untreated), model (LPS-stimulated), and FFP (LPS + 50 μg/mL FFP). Cellular metabolites were extracted and analyzed by UHPLC-Q-Exactive MS. The analytical quality was confirmed by the tight clustering of quality control (QC) samples in the principal component analysis (PCA) score plot (Figure 8A). QC samples were prepared by pooling equal aliquots of all samples and were analyzed interspersed throughout the sequence. Although QC points are not individually marked in Figure 8A, their consistent interleaving among the sample points demonstrated excellent instrument stability and data reliability throughout the acquisition process. Unsupervised PCA revealed a clear separation between the control and LPS-stimulated groups (Figure 8A), confirming that LPS induction significantly altered the metabolic profile of RAW264.7 cells, which is consistent with the establishment of a robust inflammatory model.

Crucially, the FFP treatment group was distinctly separated from the LPS model group and shifted closer to the control group in the PCA score plot. This clear metabolic shift indicates that FFP intervention effectively counteracted the LPS-induced metabolic disturbances, restoring the cellular metabolic state towards normality. To maximize the separation between groups and identify the most significant metabolite biomarkers, supervised orthogonal projections to latent structures-discriminant analysis (OPLS-DA) was employed. The OPLS-DA models showed excellent separation between the control and model groups (Figure 8B) and between the model and FFP-treated groups (Figure 8D). The validity of these models was rigorously confirmed by permutation tests (Figure 8C,E), which demonstrated that the original models were not overfitted.

**Figure 9:**
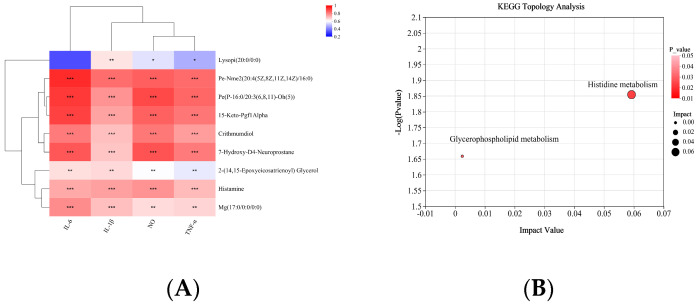
Identification, correlation, and pathway enrichment analysis of differential metabolites (DEMs). (**A**) Pearson correlation heatmap showing the relationship between differential metabolites and inflammatory mediators (NO, TNF-α, IL-1β, IL-6). Only significant correlations (|r| > 0.4 and *p* ≤ 0.05) are displayed. (**B**) KEGG pathway enrichment analysis of differential metabolites. The x-axis represents pathway impact, and the *y*-axis represents −log_10_(*p*-value). Histidine and glycerophospholipid metabolism were identified as the most significantly enriched pathways (*p* < 0.05, impact > 0.01). Statistical significance was analyzed by one-way ANOVA followed by Duncan’s test. Asterisks indicate significant differences compared to the control group: * *p* < 0.05, ** *p* < 0.01, and *** *p* < 0.001. Note: Due to the original data format provided by the analytical service, the minus sign symbols and labels represent the raw data output and could not be further modified. We have ensured that these labels are consistent with the data interpretation provided in the main text.

Using variable importance in projection (VIP) > 1.5 and *p* < 0.05 as cutoffs, we identified 9 differentially expressed metabolites (DEMs). These were upregulated by LPS and downregulated by FFP treatment to levels comparable to the control (Table 4). These key DEMs included metabolites involved in glycerophospholipid metabolism, such as dimethylphosphatidylethanolamine (DMPE) and oxidized phosphatidylethanol, as well as histamine, a critical mediator derived from histidine metabolism. To assess the functional relevance of these DEMs, we performed Pearson correlation analysis between their relative abundances and the levels of the pro-inflammatory cytokines (NO, TNF-α, IL-1β, IL-6) measured in Section 3.3. The correlation heatmap (Figure 9A) revealed that most of these DEMs, including histamine and DMPE, exhibited significant positive correlations (r > 0.4, *p* ≤ 0.01) with all four inflammatory mediators. This strong correlation suggests that the accumulation of these metabolites is closely associated with the inflammatory state of the macrophages, positioning them as potential mechanistic links between FFP treatment and inflammation suppression.

To pinpoint the most affected biological pathways, the DEMs were subjected to Kyoto Encyclopedia of Genes and Genomes (KEGG) pathway enrichment analysis. The results highlighted histidine metabolism and glycerophospholipid metabolism as the two most significantly enriched pathways (impact > 0.01, *p* < 0.05, Figure 9B). Histamine, a metabolite identified as significantly reversed by FFP treatment, has been reported to activate NF-κB signaling in macrophages. Our correlative data suggest that FFP may influence histamine-related pathways, although direct mechanistic validation (e.g., HDC activity, NF-κB activation) is required. It is well-established that histamine can activate the NF-κB signaling pathway, a master regulator of pro-inflammatory gene expression, thereby promoting the transcription and release of cytokines such as TNF-α, IL-1β, and IL-6 [30,31]. The fact that FFP treatment significantly reversed the LPS-induced upregulation of histamine provides a novel and compelling mechanistic explanation for its anti-inflammatory action: FFP likely alleviates inflammation, at least in part, by suppressing histamine production or accumulation within macrophages, thereby dampening the activation of the pro-inflammatory NF-κB cascade.

Furthermore, the modulation of glycerophospholipid metabolism, as evidenced by the reversal of DMPE levels, suggests an additional layer of regulation. Glycerophospholipids are fundamental components of cell membranes and also serve as signaling molecules [32]. Alterations in their metabolism can affect membrane fluidity, the production of lipid second messengers, and the function of membrane-associated receptors and enzymes involved in inflammatory signaling. Therefore, the normalization of glycerophospholipid metabolism by FFP may contribute to its anti-inflammatory effect by stabilizing membrane integrity and modulating signal transduction events downstream of LPS recognition.

In summary, metabolomics revealed global metabolic changes associated with FFP treatment. The data suggest that the anti-inflammatory effect of this LMW polysaccharide may involve the coordinated regulation of histidine/histamine and glycerophospholipid metabolism. However, these findings are hypothesis-generating rather than conclusive. Direct validation (e.g., targeted quantification of histamine or assessment of NF-κB activation) is required to confirm the proposed mechanism. Nevertheless, the identification of histamine as a potential downstream mediator offers a testable hypothesis for future studies on fungal polysaccharides.

### 3.5. Limitations of This Study

Several limitations of this study should be acknowledged. First, while the Caco-2 model provides evidence for trans-epithelial transport, in vivo pharmacokinetic studies are needed to confirm that FFP reaches systemic circulation after oral administration. Second, the anti-inflammatory effects were demonstrated only in vitro using RAW264.7 macrophages; animal models of inflammation (e.g., LPS-induced acute inflammation in mice) are required to validate these effects in vivo. Third, the metabolomics findings are correlative and lack targeted validation. Future studies should address these limitations by conducting animal experiments and targeted metabolite quantification.

## 4. Conclusions

This study reports the purification and characterization of a low-molecular-weight α-glucan (LMW-FFP, 6.2 kDa) from *Flammulina filiformis*. The polysaccharide was found to be directly transported across intestinal epithelial cells, primarily via macropinocytosis. It exhibited direct anti-inflammatory effects in macrophages by suppressing pro-inflammatory mediators, an activity potentially mediated through the regulation of histidine and glycerophospholipid metabolism.

Collectively, this work provides evidence for a previously less explored aspect of FFP bioactivity: the direct absorption and systemic effects of its LMW fractions. These findings advance our understanding of the structure–activity relationship of fungal polysaccharides. Specifically, LMW-FFP demonstrates direct absorption and anti-inflammatory effects. They suggest that LMW-FFP could be a valuable functional food ingredient, whose effects may extend beyond prebiotic activity to include direct immunomodulation.

## Figures and Tables

**Table 1:** Methylation analysis data for FFP.

Number of Peaks	Retention Time (min)	Derivative Name	Connection Method	Relative Molar Ratio (%)
1	16.768	1,5-di-O-acetyl-2,3,4,6-tetra-O-methyl glucitol	t-Glc*p*	18.75
2	19.786	1,4,5-tri-O-acetyl-2,3,6-tri-O-methyl galactitol	1,4-Gal*p*	0.70
3	20.080	1,4,5-tri-O-acetyl-2,3,6-tri-O-methyl glucitol	1,4-Glc*p*	67.20
4	20.498	1,5,6-tri-O-acetyl-2,3,4-tri-O-methyl glucitol	1,6-Glc*p*	1.27
5	21.963	1,3,4,5-tetra-O-acetyl-2,6-di-O-methyl glucitol	1,3,4-Glc*p*	0.77
6	23.286	1,4,5,6-tetra-O-acetyl-2,3-di-O-methyl glucitol	1,4,6-Glc*p*	11.31

**Table 2:** Chemical shift attribution of ^1^H and ^13^C of each sugar residue in FFP.

Sugar Residue		Chemical Displacement δ (ppm)						
			1	2	3	4	5	6a/6b
A	→4)-α-D-Glc*p*-(1→	H	5.25	3.47	3.8	3.52	3.68	3.61/3.69
		C	99.35	71.31	72.97	76.39	70.92	60.17
B	→4,6)-α-D-Glc*p*-(1→	H	5.21	3.48	3.8	3.51	3.68	3.72/3.78
		C	99.64	71.31	72.97	76.39	70.92	67.49
C	→6)-α-D-Glc*p*-(1→	H	4.81	3.44	3.84	3.26	3.73	3.67
		C	97.82	74.24	73.05	69.06	71.18	66.42
D	α-D-Glc*p*-(1→	H	4.8	3.42	3.69	3.25	3.88	3.61/3.69
		C	98.18	74.21	71.54	69.06	70.13	60.17
R_α_	→4)-α-D-Glc*p*	H	5.06	3.42	3.73	3.51	3.79	3.61/3.69
		C	91.73	71.26	73.02	76.38	69.25	60.17
R_β_	→4)-β-D-Glc*p*	H	4.49	3.11	3.61	3.67	3.45	3.55/3.72
		C	95.54	73.94	75.99	76.39	74.13	60.69

**Table 3:** Papp values and efflux ratio of different concentrations of FFFP.

Concentration (µg/mL)	Papp (10^−6^ cm/s)		ER
	AP→BL	BL→AP	
110	8.885 ± 0.661	4.191 ± 0.348	0.472
25	3.974 ± 0.383	1.531 ± 0.161	0.385
50	1.948 ± 0.270	0.868 ± 0.050	0.446

**Table 4:** Differential metabolites shared by model groups, control groups, and dosage groups.

Differential Metabolites	*m*/*z*	M vs.C	F vs. M
2-(14,15-Epoxyeicosatrienoyl) Glycerol	377.27	↑	↓
Pe-Nme2(20:4(5Z,8Z,11Z,14Z)/		↑	↓
16:0)	800.58	↑	↓
Mg(17:0/0:0/0:0)	365.27	↑	↓
LysoPI(20:0/0:0)	649.34	↑	↓
Histamine	112.09	↑	↓
Crithmumdiol	263.20	↑	↓
Pe(P-16:0/20:3(6,8,11)-Oh(5))	740.52	↑	↓
15-Keto-Pgf1Alpha	335.22	↑	↓

Note: “vs.” stands for “versus”. “↑” indicates that the level of the metabolite was upregulated in the specified comparison, while “↓” indicates that it was downregulated.

## Data Availability

The original contributions presented in this study are included in the article. Further inquiries can be directed to the corresponding author.

## Abbreviations

FFP	*Flammulina filiformis* polysaccharide
cFFP	crude *Flammulina filiformis* polysaccharide
LMW-FFP	low-molecular-weight *Flammulina filiformis* polysaccharide
FFFP	FITC-labeled LMW-FFP
